# Notes and Notices

**Published:** 1895-12

**Authors:** 


### NOTES AND NOTICES.

LIPPE.—On November 18th, 1895, at 7.30 A. M., at her residence, 301 D Street, Northwest, Washington, D. C., Georgeanna Lippe, widow of Dr. Constantine Lippe, of New York.

Funeral on Wednesday afternoon, at two o'clock, from her late residence.—*The Evening Star, Washington, Tuesday, November 19th*, 1895.

DUNHAM MEDICAL COLLEGE, on Wood Street, between Polk and York Streets, opposite Cook County Hospital, Chicago, was dedicated Thursday, November 14th.

The new college has the following faculty:

Theory and Practice—Temple S. Hoyne, A. M., M. D., S. Mills Fowler, M. D., Eugene W. Sawyer, M. D., H. W. Pierson, B. S., M. D., Stella E. Jacobi, M. D., J. B. S. King, M. D., R. M. Barrows, M. S., M. D., Mary Florence Taft, M. D., F. H. Lockwood, M. D., B. A. Cottlow, M. D., Frank R. Waters, M. D.

Materia Medica—Frederick O. Pease, M. D., Secretary; William O. Cheeseman, M. D., A. W. Holcombe, M. D.

Ophthalmology, Otology, and Laryngology—E. T. Allen, M. D.

Obstretrics—Hiram F. Smiley, M. D., Hubert Straten, A. B., M. D.

Surgery—C. S. Fahnstock, M. D., Howard Crutcher, M. D., Registrar; Charles W. Eaton, M. D., Hubert Straten, M. D., John D. Robertson, D. D. S.

Sanitary Science—C. D. Fairbanks, M. D.

Anatomy—F. H. Lockwood, M. D., B. A. Cottlow, M. D., A. S. Pease, M. D., Earl C. Bacon, M. D., C. E. Sayre, M. D.

Physiology, Histology, and Pathology—C. B. Stayt, M. D., Helen M. Parker, M. D., Charles J. Watts, M. D., Grant J. Gray, M. D., Frank R. Waters, M. D.

Chemistry and Toxicology—J. B. S. King, A. M., M. D., Carl Fairbanks, M. D., P. J. Latz, Ph. D., Director of Laboratory.

Medical Jurisprudence—Milton O. Naraore, LL. B.

Three months ago Dunham Medical College existed only in the fancy of half a dozen earnest disciples of Hahnemann; to-day it has one of the finest medical college buildings in Chicago, practically completed, with its laboratories and lecture-rooms fully equipped for teaching the principles and practice of medicine in the most thorough and scientific manner. It has a large and enthusiastic faculty, capable of not only teaching, but demonstrating by practice the truth of the principles upon which this college has been established.

The dedication address, by Professor William O. Cheeseman, M. D., was admirably worded and enthusiastically received. He eulogized Hahnemann, showed the material helpfulness of the two great schools of medicine. Today Homœopathy had twenty-one colleges in this country, with 2,000 students, fifty-eight hospitals, twenty medical journals, and 16,000 physicians. The mirage should become a pool, said the Scriptures, and it would prove true of this newest centre of Homœopathy.

Professor Howard Crutcher, the Registrar, made a number of announcements in a facetious vein. Some critic had written, objecting to the location of the new college, using the pronoun "we," which only sovereigns, editors, and tape-worms affected, and the speaker had not yet ceased wondering to which category belonged "this thing." (Laughter.) The Illinois State Board of Health had been notified that the college would be ready for inspection by next Monday, and the closer the inspection the better would the college be pleased. November 27th, in the amphitheatre, would be held exercises in memory of Drs. Wells and Dunham.

Professor C. S. Fahnstock, M. D., also made a very welcome address. The chaplain of the evening was Rev. W. F. Black, D. D., while the vocal selections were by Messrs. Henry Sheffield and L. Gaston Gottschalk, Marguerite Raymond and Mrs. Ada Markland Sheffield. Also Frank F. Winter and Miss May Hudson gave violin numbers. Miss Carrie R. Crane was accompanist. The artists were all honored by recalls.

Not less merry were the concluding festivities, rather all the more so, because laid in the coming dissecting-room.

How We Intend to Check Substitution of Drugs.—Owing to the fact that substitution of drugs is practiced to a great extent, we earnestly request our readers to assist us in reporting to us all cases in which they may have been the victims of this criminal offense, giving the name and address of imposters, also all particulars to substantiate their statement, such as sworn affidavit, etc.

We will expose in our pages the names of fraudulent dealers on receipt of satisfactory evidence.

All our readers will admit that a doctor who prescribes a certain remedy expects that his prescription shall be filled accordingly. A druggist has no right whatever to use his own judgment in the matter, otherwise he places the reputation of the physician as well as the life of his patient in jeopardy.

Feeling that all doctors, honest druggists, and manufacturers of legitimate preparations will be benefited by our action in this matter, we solicit their assistance.

The above notice must be considered as a warning to druggists who believe that they are at liberty to substitute drugs.

The Missing Link Found.—Dr. D. G. Brinton writes to *Science* under this title, and says that Dr. Eugene Dubois, a Dutch army surgeon stationed in Batavia, Java, has found the "link." He, the surgeon, describes three fragments of three skeletons which have been exhumed from the early pleistocene strata of Java, and which introduce to us a new species, as well as a new genus and a new family, of the order primates, placed between the Simiidæ and Hominidæ.

The material, we are told, is sufficient for a close osteological comparison. The cubical capacity of the skull is about two-thirds that of the human average. It is distinctly dolichocepalic, about seventy degrees, and its norma verticalis astonishingly like that of the famous Neanderthal skull. The dental apparatus is still of the simian type, but less markedly so than in other apes. The femora are singularly human. They prove beyond doubt, says Dr. Brinton, that this creature walked constantly on two legs, and when erect was quite equal in height to the average human male. Of the various differences which separate it from the highest apes and the lowest men, it may be said that they bring it closer to the latter than to the former.

The line of descent, according to Dr. Dubois, is: Prothylobates, Anthropopithecus sivalensis, Pithecanthropus erectus, Homo sapiens.

The discovery of Dr. Dubois leads him to think that man originated on the southern slopes of the Himalayan chain, which is, after all, not so far from the Garden of Eden, and is just south of the present abode of the mystical Mahatmas. We trust that Dr. Dubois is *homo sapiens*.—*The Medical Record.*

A Doctor's Suggestions.—Said a prominent physician to a St. Louis *Republic* writer the other day: "Why is it that authority for performing marriage ceremonies is not lodged in the hands of doctors instead of being given to the ministers? Of course, I do not advocate such a course as was taken by Lycurgus to make the people of his country a race of perfect physical beings, but there are many mild steps toward this end which might be taken by the rulers of this country. What is a minister supposed to know about whether two certain persons should marry or not? If a crippled, red-headed man, weighing 105 pounds, comes to him with a request that he be joined to a deformed, red-headed woman weighing 300 pounds, he ties the knot, waiting only long enough to examine the license and collect his fee. Now physicians have enough to do, but I believe it would be a good thing for this country and for the human race if they were given this authority, and would exercise it properly. If I had my way no two should be united for life unless they had good, strong, and sound physical make-ups. Then I would never marry two blondes, but would always require a blonde to secure a brunette for a partner. If this were done we would have fewer red-headed, weak-eyed people, and besides becoming more beautiful as a race, we would soon become stronger and longer-lived."

Reprinted from *The Journal of the American Medical Association,* June 22d, 1895.

### TREATMENT OF ASIATIC CHOLERA.

By Elmer Lee, A.M., M.D., Ph.B., Chicago.

Spasmodic cholera—called also malignant, epidemic, Asiatic, Indian, blue, and pestilential cholera—is generally epidemic, though not contagious. The first symptoms are generally experienced during the night, sometimes beginning with a light general uneasiness and moderate diarrhœa; at other times the symptoms come on violently and follow each other rapidly. In fatal cases death usually occurs at some period between six and twenty-four hours; in a few fatal cases the patient lingers two or three days. The ordinary course of symptoms are more or less diarrhœa; the discharges at first feculent, but soon presenting the appearance of rice-water or gruel; there are flying pains, or sense of coldness in the abdomen, as if purgative medicine were about to operate; the countenance is pale; there is nausea, vomiting, prostration of muscular power, and nervous agitation; cramps in the legs, arms, loins, and abdominal muscles, more or less severe; small, weak pulse, intense thirst, and urgent desire for cold water; in most cases cold, clammy skin; all these symptoms may appear successively or almost simultaneously. In some cases the premonitory symptoms exist for eight or ten days; and sometimes the patient is prostrated at once. When the disease comes on suddenly the cramps usually begin in the fingers and toes, rapidly extending to the trunk; the eyes are sunken and surrounded by a dark circle; there is vomiting and purging of white matters mixed with flocculi; the features are sharp and contracted; the expression of the countenance wild and confused. The face, extremities, and often the whole surface of the body manifest a varying intensity of a leaden, bluish or purplish hue; the extremities shrunken, the nails blue, the pulse thready or wholly imperceptible at the wrist, arm, axilla, temple or neck; there is great restlessness, incessant jactitation, severe pain in the epigastrium, loud moaning or groaning, difficult and oppressed breathing; difficult inspiration, with short and convulsive expiration; voice hoarse, whispering, or nearly suppressed and plaintive; the tongue is white, cold, and flabby, and the external temperature often sinks below 80 degrees; convulsions recur at short intervals, or a constant tremor exists. The secretions of bile, saliva, tears, and urine are entirely suppressed, and a cadaverous odor exhales from the body. The patient retains his faculties to the last.

Some of the symptoms may be disproportionately severe, or may be entirely absent. Those usually regarded as pathognomonic are: watery dejections, blue appearance of the countenance or surface, thirst, coldness of the tongue, and pulselessness at the wrist.

The foregoing description of the symptoms of cholera is indicative of the nature of the disease calling for human aid. The time in which to treat the patient sick with cholera is exceedingly limited. What is to be done must be executed with rapidity. There is not a moment to lose between the time when the patient is first seen and the accomplishment of severely practical efforts. Many wise theories may be promulgated, but there are few practical measures that will avail against Asiatic cholera. The experiences during the cholera epidemic of 1892, in Europe, both in Russia and Germany, produced in me a profound conviction that, for the most part, remedial agencies that have been used are of questionable utility. Nearly every prominent remedy proposed and tried has been found to end in greater or less dissappointment. Years ago, great reliance was placed upon the far-famed "mild chlorid of mercury." Twenty and ten years ago this remedy was given in large doses. Three years ago, during the latest epidemic, small doses prevailed. Next to this, the synthetic drug salol, the product of the laboratory of the Imperial Institute of Experimental Medicine in St. Petersburg, was the most widely used and the most favorably received. Professor Nenski, the originator of salol, personally informed me that the value of the drug could not be seriously recommended as of much importance, but that it perhaps answered the requirements as far as any drug could answer, in the hands of his colleagues. Widely circulated and various reports, enthusiastically commending and moderately commending this remedy were received by the Professor in St. Petersburg, but he himself was silent as to its efficacy. The far-famed and seemingly unmatched drug, quinin, has been used, and has been held as a dazzling gem before the eyes of the profession by some of our best men, who believe that cholora is analogous to malarial disorders, and consequently the medicine which occupies the position of keystone in the arch, for malarial treatment, is a remedy suitable to contend with the rapid and desperate symptoms of Asiatic cholera. Quinin has a stout advocate in our own country, in the person of a well-known professor in one of the Ohio medical colleges. It was not used, to my knowledge, in the treatment of cholera during the last epidemic in Europe.

A remedy was brought to Hamburg during the latter part of the epidemic of 1892 by the representative of an English syndicate, who posed as a chemist, not a physician. His remedy was a preparation of iodin, to be administered through the mouth. He called the medicine a periodate, and made some experiments upon patients in one of the cholera hospitals in Hamburg. His remedy, however, was not favorably entertained by the medical authorities in charge of the cholera patients, and whatever claims were reported came through the interest of a friendly correspondent of one of the Hamburg weekly secular papers. To show how misleading some of our supposedly authentic information often is, it is only necessary for me to refer to the report given in the "Year Book of Medical Progress," published in Philadelphia. Of all the progress made, of all the combined investigations during the entire epidemic of cholera throughout Europe in 1892, and there was an immense amount of original investigation and great effort made to discover a remedy, the curious spectacle in the Year Book, which alone refers to the remedies brought by an agent of a syndicate from London to Hamburg, at the closing of the epidemic of cholera, shows that there are some things in our profoundest medical publications that are to be taken *cum grano salis.*

Uretin was extensively used hypodermically for its alleged influence upon the secretions of the kidneys, upon the ground that the kidneys were to be aided by irritating them to greater functional activity to eliminate morbid elements through the urine. The result of many investigations recorded in Russian practice show that this drug is not to be commended. Digitalis was used, supposedly to benefit a weak heart. This remedy, if at all useful, could be little more than palliative. The use of acidulated water was extensively employed in different hospitals in Europe as a drink, but not prescribed as a remedy. The water was acidulated with HCl and $H_2SO_4$. Subcutaneous injections of salt water were made. The proportion of salt was one-half of 1 per cent., and the amount of salt water injected subcutaneously was sometimes as much as a quart at a single injection. In one instance, during an illness of several days, as much as thirteen quarts was subcutaneously injected into the cellular tissue, principally that of the abdominal wall. This process of subcutaneous injection was known as hypodermaclysis. The purpose of the hypodermaclysis was to maintain the volume of the blood. The diminished volume of the blood is directly the result of the waste of its liquid portion or serum into the alimentary canal. In this serious discharge, flakes of intestinal mucus gave the name of "rice-water discharges" to the bowel evacuations, the particles having a resemblance to grains of rice. The general inflammatory state of the intestinal mucous membrane, throughout its entirety, drains the blood of its liquid portion rapidly, and collapse due to stagnation of circulation quickly ensues.

The remedies mentioned are only a portion of those tried, but there is no living advocate who to-day can point with unerring certainty to one single organic or inorganic substance, howsoever administered, that can be safely depended upon in the treatment of Asiatic cholera. Both botany and mineralogy have been searched in vain for a cure for this disease.

The cause of this disease is perhaps accurately stated to be due to invasion of the blood and, secondarily, of all the tissues of the living organism, by toxins or ptomaines, which originate in the upper portion of the small intestine at the early stages of cholera. These products of organic activity, whether of animal or vegetable organisms it is here unnecessary to debate; but these noxious products enter the circulation through the villi of the intestine and rapidly and desperately poison the blood. It is clearly proved that the disease is the result of general blood poisoning from an intestinal origin. Whatever the chemic nature of the poison may ultimately be found to be, may be safely left to the bacteriologic laboratory. The practical and intensely important part that remains for physicians seeking to cure patients in times of this disease is to realize how much, as well as how little, it is within human power to do. The human organism is prostrated by a fierce and deadly poison. This poison is in the blood and in the cells of the tissues, and its work of destruction is quickly and effectually accomplished. Reflectively, to say nothing of experimental research, it would seem to me that the rational and only course that could be advocated with scientific assurance of relief is to, as far as possible, literally cause to be removed these products which are death-dealing to the body in which they happen to be found. Now, in this same reflective mood, think for a moment and try with me to determine whether it is possible in such conditions as produce the symptoms of Asiatic cholera, it is safer to introduce other poisonous products to neutralize the noxious elements in the blood and cells, or whether it is a better process to, without the introduction of additional foreign substances, remove what we already find in the blood. To make this proposition clearer, it could be stated in another way, namely, the body is already bearing a crushing burden; shall we add other foreign substances as an additional burden to the load already carried? The principle seems to me to be at fault. The principle is the principle of allopathy, but in the light of facts is it a safe principle to follow? It is reasonably scientific to produce in the laboratory definite results in vessels of glass by the use of fixed reagents; in the organic laboratory of the living body, no such definite results can be demonstrated. The vital principle is an entity which enters into the formula and may be represented by the unknown quantity $x$ in algebraic equations. Great and laudable efforts have been made to prevent as well as to cure this disease by inoculation.

Ferran, of Valencia, Spain, thrilled the world ten years ago with his proposition of a universal cure for this disease. His glory was then at its zenith. His fame has long since faded. So obnoxious became his proposition to the government of Spain that laws were adopted to suppress Ferran's cholera inoculations.

A worthy colleague and laborous investigator, Professor Haffkin, of Pasteur Laboratory fame, proposed a modified inoculation for the prevention and cure of cholera in 1892. A reporter of the New New *Herald* was inoculated at the Pasteur Institute, and with credentials sent to expose himself to Asiatic cholera at Hamburg in September, 1892. The same reporter had been similarly inoculated by Ferran in 1886 and had the courage to make further exploits in behalf of his newspaper, at Hamburg. A very widespread opinion prevails in America that the exploit of the New York *Herald* reporter during the ten days' stay as a nurse in the Hamburg hospital constitutes a proof of the validity of Haffkin's claim, but the scientific world of Europe knows differently. *En passant*, it may be interesting to state at this place that further experiments have been made by Professor Haffkin in India with the cholera inoculations, and, unfortunately for the proposition, reports have recently come to me from reliable medical sources, that a greater percentage are attacked with cholera who have been previously inoculated than of those who have not been inoculated. This subject of prevention, however, is to be discussed by me in a paper to be read before the Section on State Medicine.

The result of prolonged reflection, covering many years, and the observations resulting from personal experience in the cholera epidemic in Europe of 1892, is the conviction that there is provided in the laboratory of the universe a remedy which surpasses the results of human ingenuity as much as does the sun surpass in brilliancy the light of the artificial lamp. The all-pervading and all-wide remedy, the greatest product of omniscient nature's laboratory, which alone can cope with this pestilential disease of the human race, is nothing more and nothing less than the unmatched, unmatchable $H_2O$. Pure water is absolutely the only trustworthy cure for cholera, and if it came at a great price it would probably be more greatly valued. The human organism is so constituted that if it is assisted by $H_2O$, every morbid element may be eliminated out of its domain. The acutely poisoned body quickly recovers its equilibrium and its harmony of action as soon as the processes of elimination can remove the invading poison. In the construction of the mucous lining of all the accessible cavities and channels it is prepared by an undiscernible law to successfully resist the entrance of every form of organism. The products of organic action alone are able to pass into the blood. If sufficient quantities of pure water, of a suitable temperature, are introduced into the body through the natural channels, it is actually possible to wash morbid products as well as organic forms of life out of the human body. The mouth gives entrance to the causative germs in Asiatic cholera. That is quite conclusively established. The locality of the development and formation of the toxin in the earlier stages is determined to be in the upper end of the small intestine; and from experience, as well as from the powers of reflective analogy, there is no doubt that the system can be saved from death if the morbid entity, the germ, is literally deluged away from the alimentary canal by the copious use of a remedy that cannot be of the slightest danger to the victim. The amount of water to be used varies in different cases. It is impossible to use too much; it is possible to use too little. From the earliest moment that the patient is seen, the propositions should be, first, wash the whole alimentary canal with pure water; wash the lower portion by introducing irrigations of warm soapsuds or merely warm water into the colon sufficiently frequently and sufficient in quantity to cleanse that portion of the bowel effectually. The frequency of washing that portion of the bowel which is accessible from the rectum should be one, or two, or three, or four times a day, according to circumstances. At the same time from one to ten quarts of warm pure water mildly medicated with peroxide of hydrogen or hydrozone should be administered at regular intervals, during the day, as the prescribed remedy by the mouth. If the patient vomits, very well. Immediately re-introduce the quantity of water that was vomited. No harm can be done in any case, and if it is possible to save life it is possible to save it through this method. It is the quickest and the surest method of exciting the activity of the kidneys, and is the safest. It is the rational and effective measure for maintaining the volume of the blood. It is the scientific process by which to establish cutaneous circulation in the capillaries.

The use of simple and useful hygienic measures are the same as in other prostrating diseases. Patients should be fed with regularity at not too frequent intervals, giving the proper time, between administrations of simple food, for its digestion. The use of appliances for maintaining the heat of the body are not to be neglected.

The precise details of the method of treatment indicated at this time will be forthcoming in a subsequent paper.

100 State Street.

Reprint from *The New York Medical Journal*, March 2d, 1895.

### RATIONAL THERAPEUTICS OF CHOLERA INFANTUM.

By Gustavus Blech, M. D., St. Louis.

No strict rules can be given for the treatment of disease. It is for this reason that so many physicians say we do not treat a disease, but we treat an individual. True enough, we treat the individual, but what we have most of all to consider is the disease. The individual will dictate us alterations and modifications in our treatment.

A general plan of treatment may be outlined, however, and I will try to do so in regard to one of the most fatal diseases of babyhood—cholera infantum. There is a certain philosophy in therapeutics which I would frame in the three following rules: First, remove if possible the disturbing causes; second, treat symptoms which *per se* are liable to endanger the life of the patient; and third, sustain vitality.

As said before, the therapeutics, which is based upon the ætiology and pathology of a given case is the only one to be employed.

Now, the ætiology of cholera infantum is not so obscure as asserted by a good many authors. Whether or not of microbic origin, one thing is sure—it is due to a chemical decomposition of food, causing an inflammatory condition of the digestive and alimentary canal.

Clinical experience, furthermore, shows that this disease is of a grave character, producing death in a large proportion. Heat *per se* is not the immediate cause of this disease, but it influences its course considerably. Therefore, gastric or intestinal disturbances in summer demand a closer attention than those which occur during the colder season. Cholera infantum is a disease met even in the palaces of the rich, although not so often as in the tenement houses of the poor, which fact proves again that bad air, filth, and lack of ventilation are also of a predisposing influence, as well as an obstacle to a quick cure. The mortality in the tenement houses is larger than that of the richer parts.

If we consider the aforesaid, we shall, first of all, as regards the treatment of this disease, have to restrict diet.

As soon as called to a case of cholera infantum, prohibit for the first day any food whatever. Mothers have no right to nurse the little patient either. Strict instructions must be given in that direction, because the timid mothers are often inclined to quiet the crying babies by putting them to the breast.

Remedies are of very little value. Beginning with calomel, salol, and all the newer antiseptics, finishing with subnitrate of bismuth—they have all proved a failure, for none of them work quickly enough.

The treatment as outlined by Dr. Elmer Lee, of Chicago, in his cases of typhoid fever, proved a success in my hands during last summer, and under this treatment I have lost only one patient out of twenty-three, while the monuments of my skill exercised during the year 1893 are decorating the cemeteries of the State of Connecticut.

So far as I knew, the best antiseptic (which has also a strong tendency to reduce local inflammation) was peroxide of hydrogen (medicinal) until hydrozone was used by me. Hydrozone being twice as strong as Marchand's peroxide of hydrogen (for economical reasons), the latter drug is preferred by me. This remedy can be administered internally as well as externally.

I add a teaspoonful of hydrozone to a pint of water for washing out the stomach. The vomiting ceases after the first washing as a rule. If necessary, this procedure can be repeated. If the vital power of the little patient is not too low it can produce no harm. But in every case, no matter how far advanced, I do not omit an irrigation of the bowels, for which purpose I use a soft rubber catheter attached to a common bulb syringe. The catheter is introduced as high in the colon as possible. It is unnecessary to say that the water must first be sterilized. I do not agree with Dr. Lee in using hot soap water. On the contrary, I use cold water, and add to each quart about two ounces of hydrozone. The improvement after the first or second irrigation is marked. If necessary, these irrigations can be repeated every two hours.

If the fever is very high, and if the irrigation of the bowels does not reduce it, the whole body should be washed with alcohol.

The diet for the next twenty-four hours should be very light indeed. Sweet, strong Russian tea is all I allow.

Each individual case will teach us when food can be allowed again.

Since the adoption of this mode of treatment I have met with the most remarkable success, and no honest practitioner should refuse it a trial.

